# Minimally Invasive Approach for Superior Sinus Venosus Atrial Septal Defect Repair in a Child: A Case Report

**DOI:** 10.7759/cureus.98815

**Published:** 2025-12-09

**Authors:** Shamsher S Lohchab, Sandeep Singh, Panmeshwar Rathia, Shoranki Pardhan

**Affiliations:** 1 Department of Cardiothoracic Surgery, Pt. B. D. Sharma Post Graduate Institute of Medical Sciences (PGIMS), Rohtak, IND

**Keywords:** minimally invasive cardiac surgery, minithoracotomy, papvc, sinus venosus atrial septal defect, svc

## Abstract

In recent years, breakthroughs in transcatheter interventions have transformed the landscape of cardiac disease management, where less invasive techniques have gained popularity due to their aesthetic benefits. Yet, when it comes to sinus venosus atrial septal defects (ASDs), the intricate anatomical challenges have largely limited the adoption of minimally invasive surgical solutions in these cases. Reports of minimally invasive repair for this particular defect have been sparse, and most such cases involve adult patients. In this account, we detail the management of a 12-year-old child who successfully underwent correction of the defect via a right mini-thoracotomy. This technique eliminates the need for direct cannulation of the central aorta and inferior vena cava, thus maintaining a clear operative field. Repair through right mini-thoracotomy and femoral cannulation is a viable option for pediatric patients weighing at least 20 kg, providing an effective alternative to the traditional midsternotomy. Superior vena cava (SVC) drainage pre-insertion of partial anomalous pulmonary venous connection (PAPVC) was done through direct cannulation of the SVC. Notably, the distinct bay formed within the right atrium over a superiorly located defect enhances exposure to the SVC, pulmonary veins, and ASD, facilitating precise surgical intervention.

## Introduction

The surgical correction of atrial septal defects (ASDs) holds historical significance, marking the dawn of open-heart surgery after John Gibbon successfully performed the first ASD closure under cardiopulmonary bypass (CPB) in 1953. Since that landmark procedure, ASD repair has achieved outstanding outcomes, with minimal operative mortality, very low complication rates, and excellent long-term prognosis [1]. In recent years, there has been a notable shift toward minimally invasive cardiac surgery (MICS) techniques for ASD closure, motivated by their cosmetic advantages, reduced blood loss, shorter hospitalizations, and quicker patient recovery. Percutaneous device closure has also gained widespread acceptance, as it is the least invasive option and can even eliminate the need for CPB [2]. Transcatheter/percutaneous closure of sinus venosus ASD (SV-ASD) is also possible [3].

SV-ASDs arise due to the unroofing of the right pulmonary veins, which results in these veins draining anomalously into the superior vena cava (SVC) or directly into the right atrium (RA) [4]. SV-ASDs account for roughly 5%-10% of all ASD types. Diagnosis of SV-ASD on transthoracic echocardiography (TTE) is particularly challenging. It often necessitates the complementary use of more advanced studies, specifically transesophageal echocardiography (TEE), cardiac magnetic resonance imaging (CMR), cardiac computed tomography (CT), and, rarely, cardiac catheterization [5].

Owing to their intricate anatomy - often requiring complex surgical baffling or advanced devices to redirect flow from the SVC to the RA and from the right pulmonary veins to the left atrium - these defects have not been extensively managed by minimally invasive approaches [6].

In this report, we describe a successful surgical repair of a superior SV-ASD accompanied by anomalous drainage of the right superior pulmonary vein (RSPV) into the RA.

## Case presentation

A 12-year-old male child weighing 20 kg presented with chest pain during routine physical activities, persisting for two months. Upon examination, the pulse rate was 92 beats per minute and regular, the blood pressure was 110/60 mmHg, and a wide, fixed splitting of the second heart sound was noted. TTE revealed a large SV-ASD, with the RSPV and SVC straddling over the defect. There was also right ventricular volume overload without pulmonary arterial hypertension. The initial plan was to perform a routine intracardiac repair via a midsternotomy approach. However, influenced by the positive outcome of a fellow patient who underwent MICS for secundum ASD closure, the patient and his parents insisted on the MICS approach for its quicker recovery and smaller scar. Peripheral Doppler ultrasound confirmed that the right femoral artery and vein, measuring 3.5 and 6 mm, respectively, were borderline suitable.

**Figure 1:**
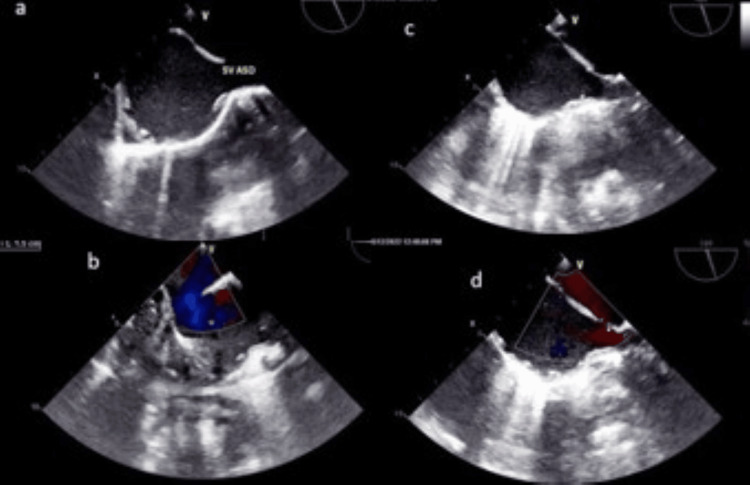
Transesophageal echocardiography. (a) Mid-esophageal bicaval view, pre-CPB 2D demonstrating SV-ASD. (b) Apical four-chamber view, probe manipulated to visualize only the atria; pre-CPB color flow mapping showing a 1.5 cm left-to-right shunt across the SV-ASD. (c) Mid-esophageal bicaval view, post-CPB 2D showing an intact interatrial septum. (d) Mid-esophageal bicaval view, post-CPB color flow mapping demonstrating no shunt across the interatrial septum. CPB, cardiopulmonary bypass; 2D, two-dimensional; SV-ASD, sinus venosus atrial septal defect

**Figure 2:**
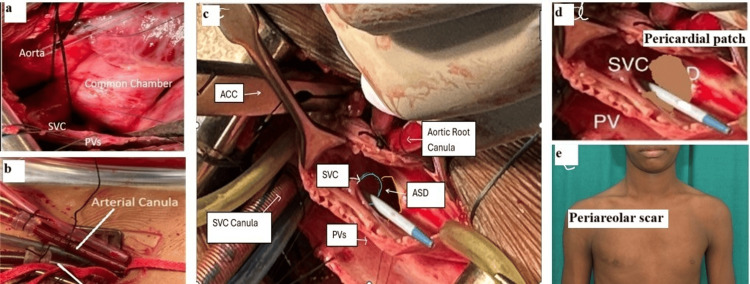
Procedural steps and cosmetic outcome. (a) External anatomy of the SV-ASD showing a well-defined chamber over the defect, distinct from the RA. (b) Femoral artery and vein cannulation. (c) Internal anatomy during cardioplegic arrest, showing the SVC and RSPV straddling a large SV-ASD, along with the SVC cannula, antegrade cardioplegia cannula, and tightened IVC tape. (d) Treated autologous pericardial single-patch baffle diverting the SVC to the RA and the RSPV to the LA. (e) Postoperative peri-areolar cosmetic scar. SV-ASD, sinus venosus atrial septal defect; SVC, superior vena cava; IVC, inferior vena cava; RSPV, right superior pulmonary vein; RA,  right atrium; LA, left atrium; ACC, aortic cross clamp

Despite the potential challenges, and with consent for conversion to sternotomy if necessary, the surgery proceeded via a right mini-thoracotomy. After the induction of general anesthesia, TEE confirmed the preoperative findings (Figures 1a, 1b). The right femoral artery and vein were exposed through a vertical 2-cm groin incision, and a right anterolateral thoracotomy (5-cm skin incision) was performed at the fourth intercostal space. A longitudinal pericardiotomy, 2 cm above the phrenic nerve, exposed the SVC, RA, RSPV, and inferior vena cava (IVC). External inspection revealed a distinct chamber in the defect region, located above the usual RA (Figure 2a).

Following heparinization, the femoral vein and artery were cannulated with 21 Fr and 16 Fr cannulas, respectively (Figure 2b). A tape was then passed around the SVC, which was subsequently directly cannulated. Antegrade cardioplegic needle insertion in the lower part of the ascending aorta was performed using a pulling stay suture. Cardiopulmonary bypass (CPB) and cardioplegic arrest using Del Nido solution were performed in the standard fashion. A tape was placed around the IVC at this stage. Vertical right atriotomy from the SVC-RA junction to the IVC-RA junction provided adequate exposure of the defect, SVC, and RSPV openings (Figure 2c). A single glutaraldehyde-treated autologous pericardial patch was sized and shaped to close the defect, incorporating the openings of both upper RSPV with continuous 5-0 polypropylene sutures (Figure 2d). Intraoperatively, before tightening the last few sutures, the openings of the pulmonary veins were visualized and found to be adequate. Similarly, the SVC opening into the RA was assessed: a 14-mm Hegar dilator passed smoothly, and the invasive gradient across the SVC-RA junction was 4 mmHg, indicating that it was not compromised. Right atriotomy was closed, and standard steps of de-airing and weaning off from CPB were followed. Protamine was administered after decannulation of the SVC, antegrade cardioplegic cannula, femoral vein, and artery. Complete hemostasis was achieved, and thoracotomy and groin wounds were closed. Routine surgical instruments were used for the entire procedure. Post-CPB TEE demonstrated no residual defect and confirmed adequate openings of the SVC and pulmonary veins (Figures 1c, 1d).

The patient was shifted to the ICU in a stable hemodynamic state and was extubated after three hours. He did not require inotropes and had minimal bleeding postoperatively. The patient made an uneventful recovery and was discharged on the third postoperative day. After 12 months of follow-up, the patient was asymptomatic, with a small peri-areolar scar mark (Figure 2e).

## Discussion

In the current era of percutaneous management for cardiac diseases, significant progress has been made in minimally invasive surgical approaches for treating secundum-type ASD. These accesses have evolved from classical sternotomy to limited sternotomy, mini-thoracotomy, and video-assisted thoracoscopy and are anticipated to advance further to robotic surgery [7-8]. Regarding the SV-ASDs with partial anomalous pulmonary venous drainage (PAPVD), there are various surgical techniques, but the main techniques that have been historically established are the two-patch technique (or baffle technique) and the Warden procedure, depending on the pulmonary drainage position. However, SV-ASD is still perceived as a complex cardiac malformation, often treated with the traditional midline sternotomy approach. Minimally invasive techniques for SV-ASD repair have been infrequently reported, primarily in adult patients [9-12].

While midline sternotomy is the conventional practice at our institute, the cosmetic and psychological concerns raised by the patient and their caregivers, coupled with the patient's young age, prompted a successful attempt to correct the superior SV-ASD with partial anomalous pulmonary venous connection (PAPVC) through a minimally invasive approach. Access was achieved through an anterolateral thoracotomy incision in a 12-year-old patient. In children, cannulation of the central aorta, SVC, and IVC is typically required, which limits space and poses challenges for the surgeon in creating a baffle for SV-ASD with PAPVC [13]. The use of peripheral femoral and venous cannulation in our patient, with right femoral artery and vein sizes of 3.5 and 6 mm, respectively, allowed unobstructed access to the operative field and provided a better view. Notably, the surgery was performed without the need for specialized instruments, relying on familiarity with routine surgical tools. Knowledge of the anatomy of crossroads formed by the right upper pulmonary vein, SVC, RA, and left atrium is key to appreciating the procedural concept.

Compared to the midsternotomy approach, the demarcated chamber of the RA over the defect was found to be more conducive for better exposure of the SVC, pulmonary vein openings, and SV-ASD.

Percutaneous device closure, while challenging, can accelerate clinical recovery compared to the gold-standard conventional open-heart surgery. The feasibility of percutaneous closure depends on precise preoperative anatomical study and real-time guidance using multimodal fusion technology [14]. Knowledge of the anatomy of crossroads formed by the right upper pulmonary vein, SVC, RA, and left atrium is key to appreciating the procedural concept. However, device closure may result in the closure of pulmonary vein openings and restricted SVC opening.

Stenosis or obstruction of the SVC is a rare complication of surgery to correct SV-ASD with PAPVD [15]. The SVC obstruction can be due to scar tissue or changes to the vein during the surgical repair, and studies report rates of obstruction ranging from 2.7% to 7.7% in patients undergoing this type of repair. Reported life-threatening complications, such as tamponade, may necessitate emergency sternotomy and CPB [16].

## Conclusions

The successful surgical repair of SV-ASD with partial anomalous pulmonary venous connection via right mini-thoracotomy and femoral cannulation in children weighing 20 kg or more avoids central cannulation, providing an unobstructed operative field. This approach addresses cosmetic concerns and offers an effective alternative to midsternotomy. Compared to the traditional midsternotomy approach, the distinctive chamber formation in the RA over the superior type of defect facilitates better exposure for visualization of the SVC, pulmonary vein openings, and SV-ASD. Surgical repair is the standard of care for a superior sinus venosus defect with partial anomalous pulmonary venous drainage. However, transcatheter closure is emerging as a safe and effective alternative in carefully selected patients. Optimal patient selection requires the integration of multimodality imaging. Three-dimensional (3-D) modeling-guided covered stent correction (CSC) of a superior SV-ASD is an alternative to surgery in selected patients; however, anatomic variations necessitate a thorough 3-D anatomic assessment before considering CSC. Heart VR is a virtual reality (VR) system that rapidly displays and renders multimodality imaging without the need for prior image segmentation. Modified techniques, along with the growing experience of interventionalists, will further enhance safety and expand the role of transcatheter closure of SV-ASD. 
